# Clinical Assessment of Fine-Tuned Open-Source LLMs in Cardiology: From Progress Notes to Discharge Summary

**DOI:** 10.1007/s41666-025-00203-x

**Published:** 2025-07-25

**Authors:** HyoJe Jung, Yunha Kim, Jiahn Seo, Heejung Choi, Minkyoung Kim, Jiye Han, Gaeun Kee, Soyoung Ko, Byeolhee Kim, Boeun Choi, Ah-Ram Kim, Jung-Min Ahn, Tae Joon Jun, Young-Hak Kim

**Affiliations:** 1 Department of Information Medicine, Asan Medical Center, 88, Olympicro 43gil, Songpagu, Seoul, 05505 Republic of Korea; 2 Department of Medical Science, AMIST, Asan Medical Center, University of Ulsan College of Medicine, 88, Olympicro 43gil, Songpagu, Seoul, 05505 Republic of Korea; 3 Division of Cardiology, Heart Institute, Asan Medical Center, University of Ulsan College of Medicine, Seoul, Republic of Korea; 4 Division of Cardiology, Department of Information Medicine, Asan Medical Center, University of Ulsan College of Medicine, 88, Olympicro 43gil, Songpagu, Seoul, 05505 Republic of Korea; 5 Department of Medical Informatics and Statistics, Asan Medical Center, University of Ulsan College of Medicine, Seoul, Republic of Korea

**Keywords:** Medical documentation, Large language model, Synthetic medical records, Discharge summary

## Abstract

The generation of accurate discharge summaries from clinical progress notes represents a critical challenge in healthcare documentation, particularly in specialized domains like cardiology where limited annotated data and complex medical terminology pose significant barriers to automation. To address this challenge and improve clinical workflow efficiency, we developed a comprehensive approach combining synthetic data generation with fine-tuned large language models (LLMs), specifically leveraging Llama3.1-8B for automated discharge summary creation. Our methodology involved constructing a hybrid dataset by combining 4658 real-world cardiology discharge summaries with 12,661 high-quality synthetic records generated via the OpenAI API and validated through a T5-based binary classifier that filtered out low-quality outputs. The fine-tuned Llama3.1-8B model demonstrated superior performance across multiple evaluation metrics including ROUGE, BLEU, and BERTScore, while qualitative assessment by three expert cardiologists confirmed the model’s ability to generate clinically coherent, complete, and medically relevant discharge summaries with high accuracy in capturing patient conditions and treatment details. This research makes significant contributions to the healthcare informatics community by demonstrating the feasibility of using fine-tuned open-source LLMs for specialized clinical documentation tasks, establishing a validated framework for synthetic medical data augmentation in low-resource scenarios, and providing evidence that AI-assisted clinical documentation can achieve both technical excellence and clinical utility, thereby offering a scalable solution to reduce administrative burden on healthcare professionals while maintaining high standards of patient care documentation.

## Introduction

Discharge summaries are a foundation of healthcare communication [1], offering a detailed summary of a patient’s hospital stay, including diagnoses, treatments, and follow-up care instructions. These documents are critical for ensuring smooth transitions between care settings, improving communication among healthcare providers, and facilitating effective patient management post-hospitalization [2]. Moreover, discharge summaries play a significant role in reducing readmission rates by enabling the proper management of ongoing care plans, thereby ensuring the continued quality and safety of patient care [3–5].

With the advancement of AI, particularly natural language processing (NLP) technologies, there is growing interest in applying AI across various domains, including healthcare [6]. NLP algorithms have demonstrated remarkable capabilities in automating tasks that require understanding and generating human language [7]. In healthcare, these applications range from automating clinical documentation to improving patient-provider interactions through conversational agents.

LLMs have gained significant traction in the medical field, with applications such as generating clinical notes, interpreting lab results, and anonymizing patient data [8]. Their ability to generate human-like text offers the potential to improve both the efficiency and accuracy of medical documentation [8]. By reducing the administrative burden on healthcare professionals, LLMs enable them to focus more on patient care. However, implementing LLMs in healthcare presents challenges, particularly with regard to data scarcity, linguistic diversity, and the specialized medical terminology encountered in multicultural hospital settings [9, 10]. This complexity requires LLMs to accurately interpret medical terminology across various languages and understand the nuanced language of clinical communication.

To address these challenges, our study proposes a novel approach that combines synthetic data generation with specialized LLMs implementation. Using the OpenAI API and a T5-based binary classifier [11], we developed a method to create and validate synthetic medical records, following recent practices in AI-assisted clinical text generation [12]. Although the generated data was de-identified, we recognized the sensitivity of medical information and took additional precautions to mitigate any potential risks [13]. Specifically, we favored the use of locally trained, open-source models over cloud-based APIs, ensuring that patient data remains within secure environments and minimizing the risk of data breaches or unauthorized access.

Our study highlights the potential of deploying specialized LLMs, specifically Llama3.1-8b, to automate the creation of discharge summaries within cardiology. By leveraging both real-world hospital patient records and carefully curated synthetic data, Llama3.1-8b enhances the efficiency and continuity of medical documentation. Supported by cardiology professionals for its clinical relevance and utility, this model represents a promising solution for improving patient care through streamlined medical documentation. This advancement marks a significant step forward in integrating AI-driven tools into clinical practice, enhancing the accuracy and efficiency of medical record creation directly at the point of care.

## Related Works

The application of NLP in the medical field has been experiencing a steady increase [14–18]. This trend is reflected in numerous studies and projects that leverage NLP techniques to extract valuable insights from medical texts, improve patient care, and facilitate medical research [19]. As such, our work contributes to this growing body of research, further demonstrating the potential of NLP in healthcare.

### LLMs Application in Medical Domain

Recent advancements in LLMs have demonstrated transformative potential across diverse medical applications, spanning clinical note generation, medical question answering, patient data de-identification. These innovative approaches leverage advanced NLP techniques to address complex challenges in healthcare.

Clinical note generation [12, 20] using LLMs focuses on automating medical documentation by extracting and synthesizing patient information from electronic health records (EHRs). Recent studies demonstrate LLMs’ capability to generate contextually accurate clinical notes that capture complex medical narratives, employing advanced techniques like fine-tuned transformer architectures and domain-specific medical knowledge integration [21]. These models aim to reduce physician documentation time, standardize medical record formats, and minimize transcription errors, while addressing critical challenges such as maintaining patient privacy, ensuring clinical accuracy, and mitigating potential model hallucinations through rigorous validation frameworks.

Medical question-answering systems represent another critical area of LLMs application [22, 23]. By integrating vast medical knowledge bases with contextual understanding, these models provide sophisticated clinical decision support tools. They can synthesize patient symptoms, medical histories, and the latest research literature to offer nuanced, context-aware insights. Such systems not only assist healthcare professionals in diagnostic reasoning [22] but also enable personalized patient education and preliminary health consultations.

Patient data privacy remains a paramount concern, and LLMs has emerged as powerful tools for medical text de-identification [24]. Advanced models can automatically detect and remove personally identifiable information (PII) from electronic medical records, clinical notes, and research documents. Unlike traditional rule-based and machine learning approaches [25–28], these using LLMs methods employ contextual understanding to ensure comprehensive anonymization while preserving the semantic integrity of medical narratives.

Despite these promising developments, challenges persist. Ethical considerations, potential model biases, data privacy concerns, and the need for robust validation remain critical areas of ongoing research. Future work must focus on enhancing model transparency, improving generalizability, and ensuring responsible AI deployment in sensitive medical contexts.

### Synthetic Medical Data Generation

Recent advancements in deep learning have introduced a new paradigm in medical data generation. In particular, Generative Adversarial Networks (GANs) [29] and LLMs play a crucial role in enhancing the diversity and realism of synthetic medical data. GANs, leveraging the adversarial training between a generator and a discriminator, have demonstrated their capability to produce high-quality data. They have been extensively applied in various domains, such as generating medical images (e.g., CT, MRI, and pathology slides) [30], augmenting datasets for rare diseases [31], and simulating patient scenarios [32]. Compared to traditional data augmentation techniques, GAN-based approaches excel at capturing the intricate and complex features of medical data, offering more realistic and nuanced synthetic datasets.

LLMs has opened new possibilities for generating text-based medical data. For example, models like GPT can generate or supplement textual data such as clinical notes, diagnostic reports, and patient summaries. These models leverage context and patterns learned from original datasets to produce text with high grammatical accuracy and contextual relevance. Notably, LLMs enhance the diversity and richness of medical text data by capturing subtle linguistic patterns found in patient records, thereby supporting broader applications in healthcare research.

Despite their potential, the application of GANs and LLMs in medical data generation presents several challenges. First, ensuring that the generated data accurately represents the underlying characteristics of the original data remains a complex task. Without robust validation, synthetic data risks introducing inaccuracies that could lead to flawed medical decisions. Second, there are concerns about the potential leakage of sensitive information during the data generation process. Addressing this requires advanced techniques such as differential privacy and additional anonymization steps after data generation.

### Supervised Fine-Tuning (SFT)

Supervised fine-tuning (SFT) is a widely used approach to adapt general-purpose large language models (LLMs) to domain-specific downstream tasks. In the clinical NLP field, SFT has been effectively applied to tasks such as medical question answering and clinical note summarization, where paired training data allows the model to align its output distribution with structured targets [33, 34]. SFT typically involves minimizing a token-level cross-entropy loss between the model’s predictions and reference texts. In this study, we employ standard SFT techniques to fine-tune a general LLM using synthetic discharge summary data, following precedents in clinical summarization research [35, 36].

### Parameter-Efficient Fine-Tuning (PEFT)

To reduce computational costs and memory requirements, parameter-efficient fine-tuning (PEFT) methods such as LoRA have been proposed [37]. PEFT modifies only a small subset of parameters within large models—typically linear projections in attention layers—while keeping most weights frozen. This approach has demonstrated comparable performance to full fine-tuning in various biomedical NLP settings, including named entity recognition and clinical classification [38]. In our setting, we apply QLoRA, a quantized variant of LoRA, to enable efficient training on large models such as LLaMA3.1-8B using limited hardware resources [39].

## Methods

### Dataset

#### Ethical Approval

This study’s protocols received approval from the Asan Medical Center Institutional Review Board (IRB No.2023-1001), aligning with the principles outlined in the 2008 Declaration of Helsinki. Moreover, the need for informed consent was waived due to the utilization of an anonymous, de-identified database for research purposes. To enhance data security, only a subset of de-identified data was used with external APIs for synthetic data generation, while the main model development and evaluation were conducted using local models to minimize any residual risk of data leakage or re-identification.

#### Dataset Description

**Fig. 1:**
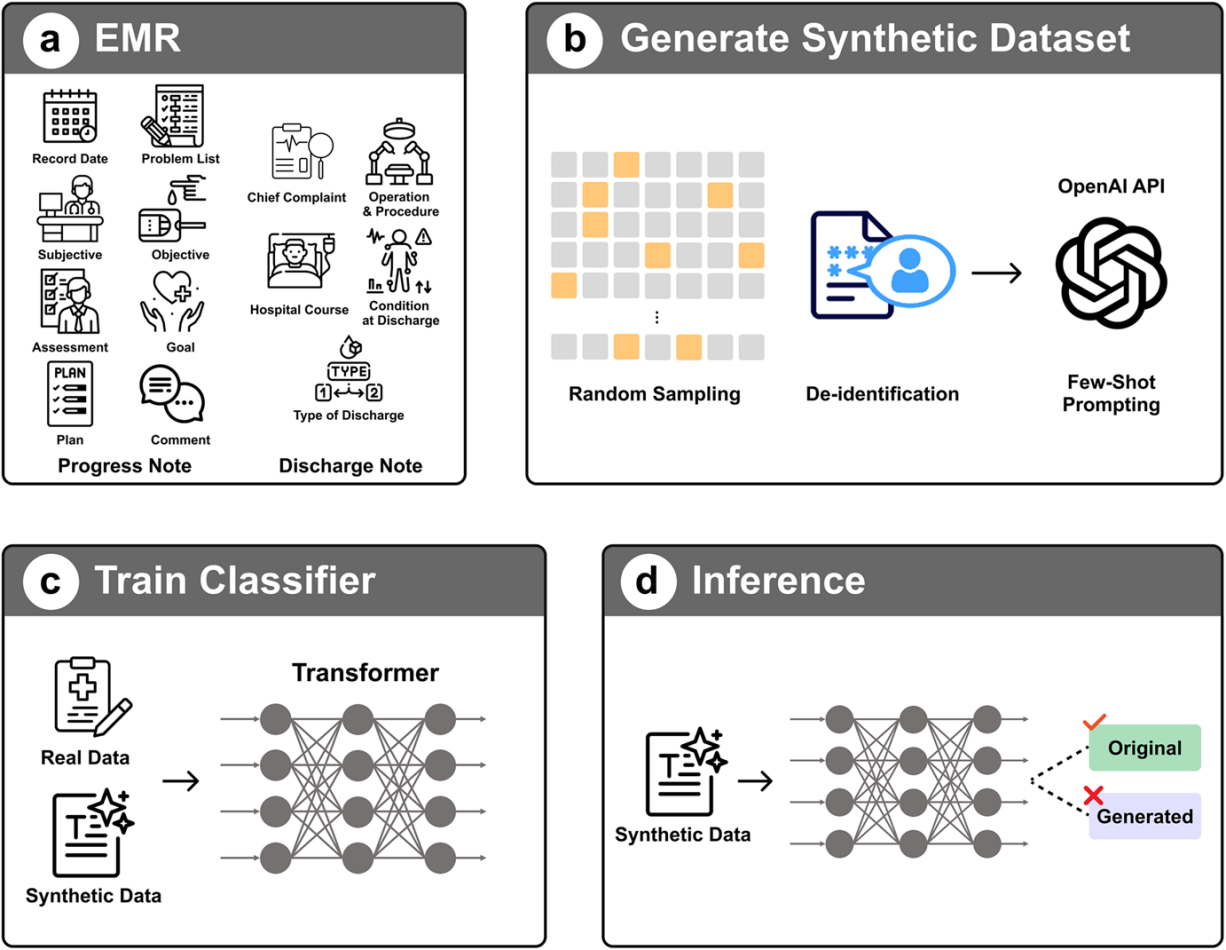
Generating synthetic data with EMR data

The dataset (Fig. 1) used in this study was sourced directly from CardioNet [40], ensuring consistency and comparability with prior research in the field. This dataset provides a rich and comprehensive collection of patient records spanning from September 2018 to December 2021, enabling in-depth analysis of various clinical parameters over time.

In this research, the Progress Notes documenting the detailed course of patient treatment were utilized as the input data for our model, while the Discharge Summaries reflecting the patient’s status at discharge served as the target output. The Progress Notes include fields such as Record Date, Problem List, Subjective, Objective, Assessment, Goal, Plan, and Comment, offering a granular view of the clinical process. The Discharge Summaries contain structured sections like Chief Complaint, Operation and Procedure, Hospital Course, Condition at Discharge, and Type of Discharge, providing a comprehensive summary of each patient’s hospital journey. This input–output alignment offers a solid foundation for LLM-based discharge summary generation.

#### Data Preprocessing and Augmentation

For computational efficiency and compatibility with the model’s input length constraints, we selected a subset of unique patient records (*n* = 4658), each with a tokenized length below 2048 tokens. While this subset ensured technical feasibility, it was insufficient for effectively training large-scale language models. To address this limitation and enhance data coverage, we implemented a two-phase data augmentation pipeline.

Approximately 50,000 synthetic records were generated using the OpenAI API and subsequently filtered through a T5-based binary classifier trained to distinguish between real and synthetic discharge summaries. Only those classified as “original” (*n* = 12,661) were retained and combined with the initial 4658 real records to construct the final dataset. Although the classifier was trained on a balanced dataset, no formal validation metrics (e.g., accuracy, precision) were reported, as the model was not evaluated on a held-out validation set. Instead, a conservative filtering strategy was adopted to maximize the reliability of selected synthetic records.

1. Synthetic Data Generation
  We utilized the OpenAI API to generate synthetic medical records based on de-identified samples from our original dataset. This process involved carefully extracting key structural and linguistic characteristics from authentic medical records, which were then used as prompts to generate additional synthetic documents. The synthetic generation process was meticulously controlled to ensure that the generated records maintained the essential medical terminology, clinical narrative structure, and contextual nuances of the original documents. The OpenAI model was selected due to its strong performance in natural language generation, particularly its ability to produce coherent and fluent clinical narratives with fast inference speed and high reliability, making it well-suited for scalable synthetic data generation in this study.
2. Synthetic data validation
  To validate the quality of our synthetically generated medical records, we developed a robust T5-based binary classifier. The classifier was trained on a balanced dataset of original medical records and synthetic records, enabling it to distinguish between authentic and generated documentation with high precision.
    During the validation phase, we applied this classifier to the entire generated dataset, selecting only those synthetic records classified as “original.” This rigorous filtering process ensured that our final augmented dataset maintained the linguistic patterns, structural coherence, and contextual depth characteristic of authentic medical documentation, thereby enhancing the reliability of our research data.

Following the two-phase generation and validation process, the resulting dataset—comprising filtered synthetic records (*n* = 12,661) and original records (*n* = 4,658)—was randomly shuffled and partitioned into training samples (*n* = 15,144), validation samples (*n* = 461), and test samples (*n* = 1714). This split ratio was chosen to ensure representative sampling, minimize bias, and maintain a balanced distribution for effective model training and evaluation.

### Models

**Fig. 2:**
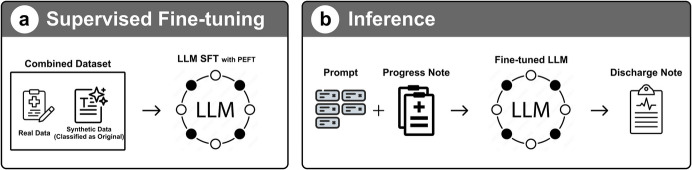
Supervised fine tuning with combined dataset

In our comprehensive study of automated discharge summary generation, we employed a sophisticated approach utilizing a diverse array of LLMs and a specialized binary classifier. Our methodology centered on supervised fine-tuning (SFT) (Fig. 2) to adapt pre-trained models to the intricate linguistic demands of medical documentation.

We utilized five prominent LLMs: Llama3.1-8B [41], Llama3.1-70B [41], Mistral-Small, Mistral-Nemo, and Qwen2.5-32B, each selected for its potential to effectively process and synthesize complex medical narratives. Concurrently, we developed a T5-based binary classifier as a critical component of our data augmentation strategy. This classifier was purpose-built to validate the authenticity of medical documentation, trained on an equally distributed dataset of original and generated medical records.

### Algorithm Overview

The overall pipeline for discharge summary generation consists of four main stages: synthetic data generation, filtering, model training, and evaluation. As shown in Algorithm 1, we begin by generating progress notes and discharge summaries using a general-purpose language model (OpenAI API) prompted with structured input extracted from de-identified clinical records. To ensure data quality, a T5-based binary classifier is applied to retain only summaries that are semantically close to original samples.

**Algorithm 1:**
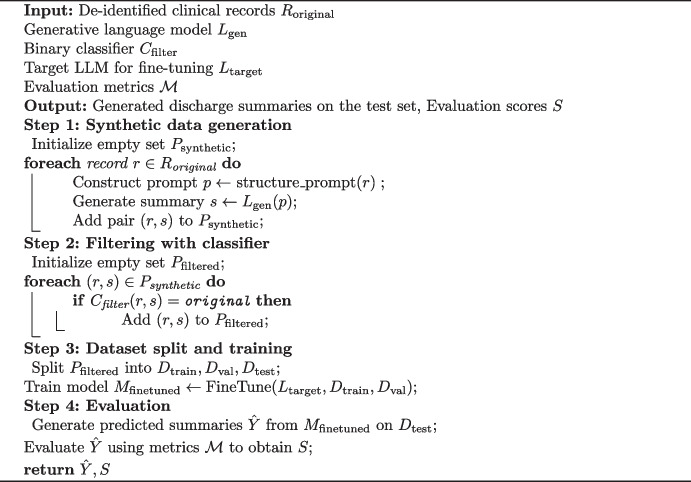
Discharge summary generation pipeline

The filtered dataset is then split into training, validation, and test sets, which are used to fine-tune a domain-agnostic large language model (e.g., Llama3.1-8B). Finally, the fine-tuned model is evaluated using commonly accepted automatic metrics such as ROUGE, BLEU, and BERTScore, where the test set serves as unseen clinical input for summary generation. This pipeline ensures both control over data quality and adaptability to downstream model refinement.

### Implementation details

All models were implemented using the Hugging Face Transformers and PEFT libraries. Training was conducted on an NVIDIA A6000 GPU using bfloat16 precision. We used the AdamW optimizer with a learning rate of 2e-5, batch size of 4, and trained for 3 epochs with linear learning rate decay. The maximum token length was set to 2048 tokens for both input and output.

For parameter-efficient fine-tuning, we applied QLoRA with a rank of 8 and an alpha scaling fac- tor of 16. The target modules included “k”, “q”, “v”, and “gate”, which are the key linear projection layers within the self-attention mechanism of transformer-based architectures. These modules were selected based on standard configurations adopted in open-source QLoRA implementations. They represent commonly used injection points for low-rank adaptation, enabling efficient fine-tuning without full model retraining. This setup balances performance and resource efficiency while maintaining compatibility with existing model architectures.

## Results

In our study, we employed both quantitative and qualitative evaluations to assess the model performance. For the quantitative evaluation, we utilized several common metrics.

- Recall-Oriented Understudy for Gisting Evaluation (ROUGE): ROUGE is a set of metrics used for evaluating automatic summarization and machine translation. It compares the output with reference summaries to measure the quality, focusing on the overlap of n-grams, words, or bytes.
- Bilingual Evaluation Understudy (BLEU): BLEU is a metric for evaluating a generated sentence to a reference sentence. It calculates the precision of n-grams in the generated sentence that also appear in the reference sentence, offering a quantitative measure for translation quality.
- BERTScore: BERTScore is a metric for evaluating text generation tasks by calculating the similarity of token embeddings between the generated and reference texts. It leverages the BERT model’s ability to capture complex semantic representations, providing a more nuanced evaluation.

These measures allowed us to objectively evaluate the performance of our model in terms of various aspects such as precision, recall, semantic coherence, and language fluency. Specifically, ROUGE metrics are recall-oriented and capture how much content from the reference is preserved; BLEU emphasizes precision by measuring the proportion of correctly generated n-grams; and BERTScore evaluates semantic similarity using contextual embeddings, thus providing insight into coherence and meaning beyond surface-level matches.

On the other hand, for the qualitative evaluation, we obtained expert judgement from the Cardiologists. This allowed us to incorporate a professionals’ assessment into our evaluation process, providing a more comprehensive and practical perspective on the usability and accuracy of our generated content. The evaluation was conducted based on the following five criteria.

- Accuracy: Evaluates how accurately the generated discharge summary reflects the patient’s actual medical condition, treatment process, and recommended follow-up actions.
- Completeness: Assesses whether all important medical information (diagnosis, treatment methods, observations of improvement or deterioration, discharge criteria, and follow-up actions) is included in the discharge summary.
- Readability and Comprehensibility: Evaluates whether the generated document is easy to understand and clear for the target audience (patients, guardians, other medical professionals, etc.).
- Consistency: Assesses whether the discharge summaries generated across various patient records maintain a consistent format and quality.
- Utility: Evaluate whether the documentation generated is specifically helpful in making clinical decisions and contributes to the development of follow-up care plans.

### Quantitative Result

**Fig. 3:**
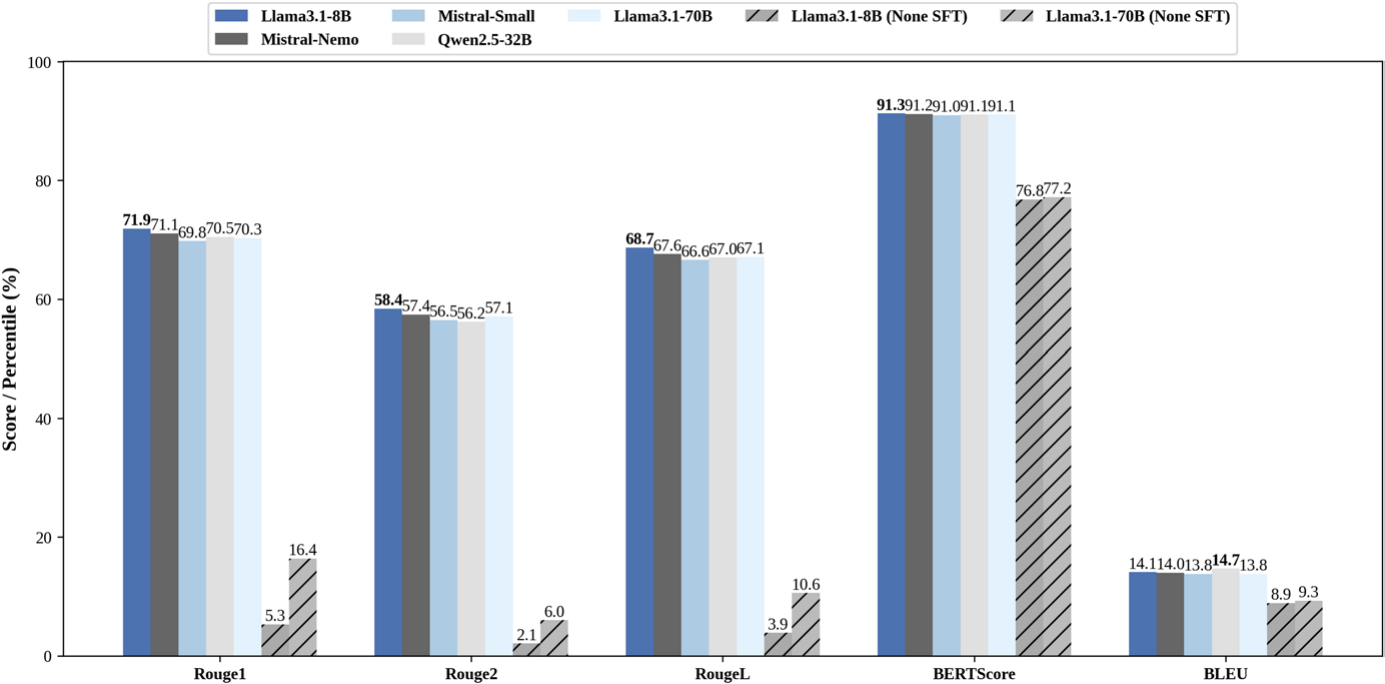
Performance of each fine-tuned model

Figure 3 presents the evaluation results of various fine-tuned language models on the task of generating discharge summaries for cardiac patients, using multiple quantitative metrics.

Notably, Llama3.1-8B demonstrated superior performance across most metrics, highlighting the potential of fine-tuned language models to generate accurate, coherent, and clinically relevant discharge summaries from patient medical records.

### Qualitative Result

**Fig. 4:**
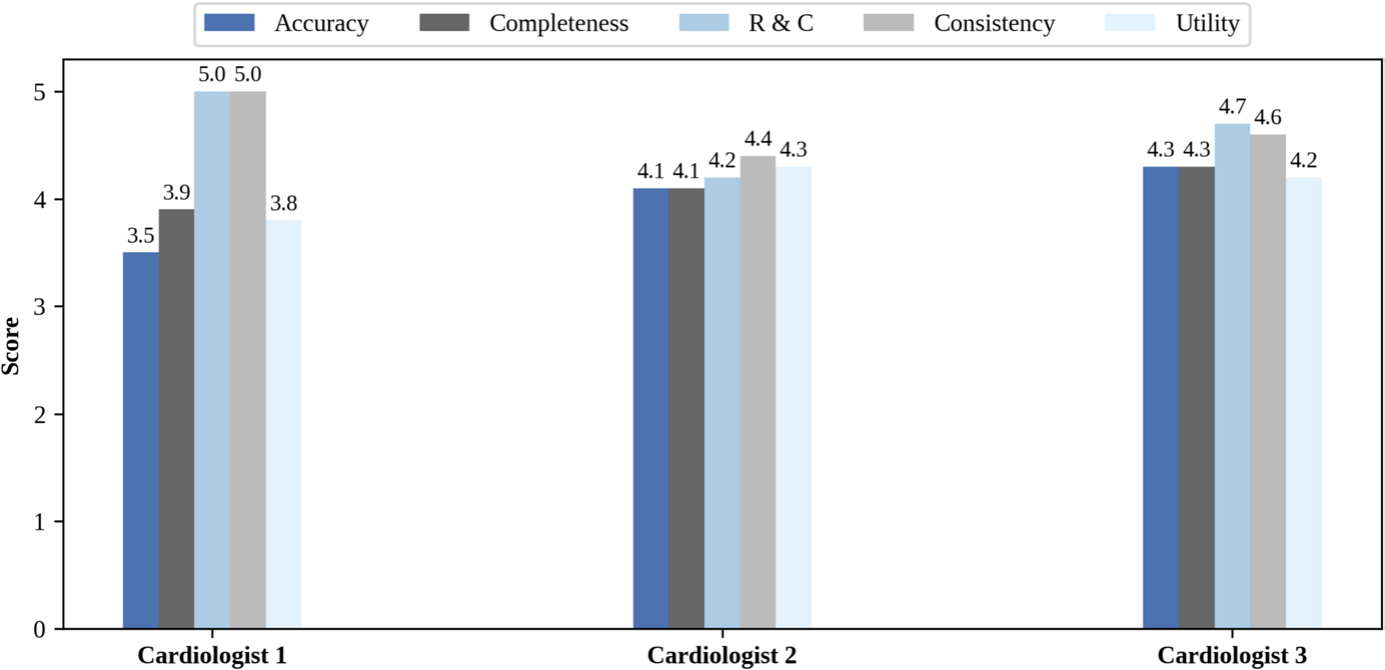
Qualitative assessment of generated discharge summaries by SFT-Llama3.1-8B

The qualitative assessment of the discharge summaries generated by the Llama3.1-8B model is presented in Figure 4. Ten sample notes from the test set, selected based on representative and high-quality outputs, were evaluated by each cardiology expert across five criteria: Accuracy, Completeness, Readability and Comprehensibility, Consistency, and Utility, with each aspect rated on a 5-point scale.

All three cardiologists independently evaluated the same set of 10 discharge summaries. The evaluation was conducted in a blinded setting, where the origin of each note—whether real or generated—was not disclosed to the reviewers in order to minimize potential bias.

**Fig. 5:**
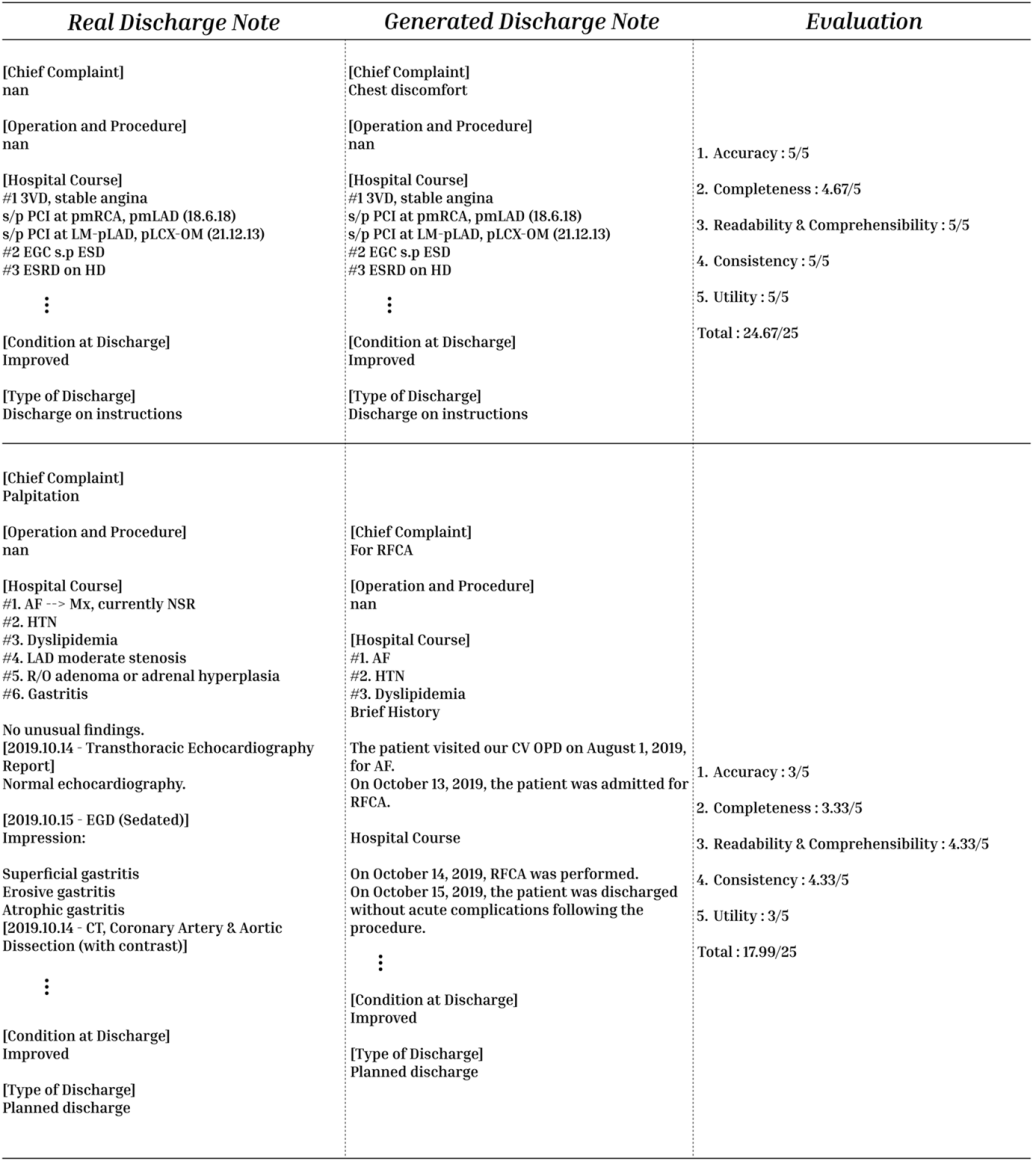
Generated discharge summaries by Llama3.1-8B and qualitative evaluation (conjunctions, words, etc., written in Korean are translated into English)

The medical record review conducted by experts revealed a documentation approach that, while capturing essential clinical information, demonstrated significant opportunities for improvement in comprehensiveness and precision. The current documentation style reflects a basic reporting mechanism that captures procedural outcomes and key events, but falls short in providing a nuanced, holistic narrative of the patient’s medical journey. The review suggests a need for a more sophisticated documentation strategy that not only records medical interventions but also contextualizes them within a broader clinical framework. By integrating more detailed decision-making processes, comprehensive patient history, and clear forward-looking treatment plans, the medical documentation can evolve from a mere transactional record to a more meaningful, patient-centered communication tool that enhances overall medical care quality and interdisciplinary understanding (Fig. 5).

## Discussion

The findings of this study emphasize the significant potential of fine-tuned LLMs, particularly Llama3.1-8B, in revolutionizing healthcare documentation practices, specifically in automating the generation of discharge summaries for cardiac patients. The quantitative results, evaluated across multiple metrics such as ROUGE, BLEU, and BERTScore, collectively demonstrate the models’ ability to generate accurate, coherent, and clinically relevant discharge summaries from patient medical records.

While the quantitative metrics provide valuable insights, they are not specifically designed to evaluate medical documentation, which poses a significant challenge in comprehensively assessing the generated discharge summaries’ quality. These metrics may not fully capture critical aspects of healthcare documentation, such as adherence to clinical guidelines, appropriate use of medical terminology, and patient safety considerations. Moreover, the lack of standardization in progress note formatting and style introduces additional complexity. Physicians often write notes in a free-form manner, leading to inconsistencies in date formatting, abbreviations, and terminology usage that can potentially impact the model’s learning process. The qualitative assessment by an expert in cardiology reinforces the practical utility of the generated discharge summaries. The Llama3.1-8B model exhibited a high degree of accuracy in capturing patients’ medical conditions, treatment processes, and follow-up recommendations. The generated summaries were deemed complete, maintaining excellent readability and comprehensibility for patients, guardians, and healthcare professionals. The consistent quality across various patient records and the potential to support clinical decision-making highlight the model’s promising real-world implementation prospects.

To address current limitations and advance this research, several key directions emerge. Firstly, developing domain-specific, clinically oriented evaluation metrics is crucial. These metrics should comprehensively assess adherence to clinical guidelines, medical terminology usage, and patient safety considerations. Secondly, extending this approach to other medical specialties beyond cardiology could broaden the applicability of AI-driven documentation. By expanding the dataset to include diverse medical conditions and specialties, the models could be fine-tuned to generate discharge summaries tailored to specific field requirements. However, while synthetic data augmentation was essential to overcome data scarcity, we acknowledge its potential limitations. Generated summaries, though filtered by a classifier, may still reflect stylistic or semantic biases introduced by the generative model. These artifacts could propagate into downstream model behavior and affect generalizability. Future work should include human evaluation or domain-specific constraints during generation to mitigate such risks. In parallel, future studies should consider expanding the scope of expert evaluation. In this study, three cardiologists independently assessed the discharge summaries in a blinded setting, without knowledge of whether each note was real or synthetically generated. This design helped minimize potential bias during evaluation. However, inter-rater agreement metrics such as Cohen’s Kappa [42] were not reported due to the small sample size (*n* = 10) and limited variance in Likert-scale responses, which constrained statistical interpretability. To ensure more robust qualitative validation, future work should involve larger expert panels and incorporate formal agreement measures to better assess consistency among reviewers.

Further research should focus on enhancing the models’ generalizability by incorporating data from multiple healthcare facilities and diverse patient populations. The exploration of multi-modal approaches presents a particularly promising avenue. By integrating textual data with medical images, lab results, and other patient information, models could develop a more holistic understanding of patient conditions. Advanced techniques like attention mechanisms and multimodal fusion could significantly improve the models’ ability to generate comprehensive and accurate discharge summaries. However, it is worth noting that the training dataset was filtered to include only records with fewer than 2048 tokens due to model input limitations. While this enabled stable and efficient training, it may constrain the model’s capacity to generalize to longer and more complex discharge summaries commonly encountered in real-world clinical practice. In addition, although not explicitly explored in this study, the automated generation of discharge summaries has significant potential to support clinical coding workflows by providing standardized, structured documentation that facilitates accurate coding and billing. Recent research has further emphasized the importance of leveraging AI models in clinical coding pipelines to enhance administrative efficiency and data quality [43].

## Conclusion

This study demonstrates the feasibility and effectiveness of leveraging LLMs, specifically Llama3.1-8B, to generate discharge summaries from structured medical records in the cardiology domain. By integrating synthetic data generation and classifier-based validation, we addressed the challenge of limited annotated data and constructed a high-quality training corpus. The fine-tuned model achieved strong performance across multiple evaluation metrics and received favorable qualitative assessments from domain experts, highlighting its clinical relevance and practical utility.

Despite these promising results, we acknowledge the limitations of relying on synthetic data and domain-specific biases, which may affect generalizability. Future work should explore multi-institutional datasets, specialty-specific fine-tuning, and human-in-the-loop evaluation to ensure robustness and applicability in diverse clinical settings. This research contributes to the growing body of work on AI-assisted clinical documentation and provides a foundation for further exploration of LLMs in real-world healthcare environments.

## Data Availability

No datasets were generated or analysed during the current study.
